# The Function and Therapeutic Implications of TNF Signaling in MDSCs

**DOI:** 10.3390/biom12111627

**Published:** 2022-11-03

**Authors:** Kun Yu, Chengxin Yu, Liping Jiao, Kun Miao, Li Ni, Xiaoquan Rao, Ling Zhou, Chunxia Zhao

**Affiliations:** 1 Division of Cardiology, Departments of Internal Medicine and Hubei Key Laboratory of Genetics and Molecular Mechanisms of Cardiological Disorders, Tongji Hospital, Tongji Medical College, Huazhong University of Science and Technology, Wuhan 430030, China; 2 GI Cancer Research Institute, Tongji Hospital, Tongji Medical College, Huazhong University of Science and Technology, Wuhan 430030, China; 3 Shanxi Bethune Hospital, Shanxi Academy of Medical Sciences, Tongji Shanxi Hospital, Third Hospital of Shanxi Medical University, Taiyuan 030032, China

**Keywords:** tumor necrosis factor α (TNF-α), myeloid-derived suppressor cell (MDSCs), tumor, inflammation

## Abstract

Myeloid-derived suppressor cells (MDSCs) are a group of immature and heterogeneous myeloid cells with immunosuppressive functions. MDSCs play important roles in the pathogenesis of cancer, chronic inflammatory diseases, and many autoimmune disorders. The accumulation and activation of MDSCs can be regulated by tumor necrosis factor α (TNF-α). In this review, we summarize the roles played by TNF-α in the recruitment, immunosuppressive functions, and chemotaxis of MDSCs, and discuss the potential therapeutic effects of TNF-α upon these cells in tumor growth and some inflammatory disorders.

## 1. Introduction

Inflammation is a process that alerts an organism’s immune system to invasion by pathogens, injuries to tissues, and other attacks. It is also an essential process in the elimination of pathogens and the repair of damaged tissues. However, organisms can be injured by uncontrolled inflammation or constant low-grade inflammation. A balance of inflammation and immune system processes is important, both to maintain normal physiological functions and to promote recovery from disorders [1].

Tumor necrosis factor alpha (TNF-α), a key cytokine linking inflammation and the immune system, can be produced by a variety of cell types throughout living organisms [2]. Anti-TNF therapy has become a mainstay treatment for some autoimmune diseases [3,4,5]. TNF is primarily produced by activated macrophages, NK cells and T lymphocytes. Therein, TNF mainly produced by macrophages is named TNF-α while that chiefly produced by T lymphocytes is named TNF-β [6,7]. They share about 30% sequence identity and have common receptors [8]. However, experimental evidence indicates that it is TNF-α rather than TNF-β that primarily activates MDSCs, resulting in the expansion of MDSCs, as well as enhancing chemotaxis and the suppressive capacity of these immune suppressors, which further promotes the occurrence and development of a variety of diseases such as tumors and some chronic inflammatory diseases [9,10]. Therefore, the regulation of TNF-α in MDSCs may provide new options for the treatment of such disorders.

## 2. The Physiological Effects of TNF-α and Its Receptors

TNF-α exists in two forms: transmembrane TNF-α (tmTNF-α) and soluble TNF-α (sTNF-α). tmTNF-α is expressed in the form of type II transmembrane peptides composed of 233 amino acids (26 kDa) on the surfaces of activated macrophages, lymphocytes, etc. It is then processed by the TNF-α-converting enzyme and converted to sTNF-α, which contains 157 amino acids (17 kDa). Both forms of TNF-α can be activated through forming trimers [7]. However, different forms of TNF-α produce differing or even opposing biological results.

TNF-α exerts effects by binding to its receptors: TNFR1, and TNFR2 [11]. TNFR1 is expressed on virtually all cell types and can be activated by sTNF-α and tmTNF-α. TNFR2 is predominantly expressed on immune cells, and its main ligand is tmTNF-α [7,12]. sTNF-α and tmTNF-α preferentially trigger TNFR1 and TNFR2, respectively [13]. These two receptors have similar cysteine-rich extracellular substructures. However, there is no homology in their intracellular domains, and they activate different downstream signaling pathways [14,15]. TNFR1 acts as a death receptor, its cytoplasmic fractions include death domains [16]. Inflammation and programmed cell death associated with tissue damage activated by TNF-α are mostly mediated by TNFR1 [14]. TNFR2 does not contain death domains [17]. Several studies have demonstrated that TNFR2 can greatly promote the activation, migration, and proliferation of cells, as well as mediating the signaling pathways facilitating tissue repair and angiogenesis [14,17].

In summary, TNF-α can be seen as a double-edged sword. On the one hand, it is involved in maintaining the homeostasis of the immune system and the defenses of the host. On the other hand, TNF-α participates in the pathological processes of malignancies and autoimmune diseases [8,18]. Recent studies show that TNFR2 expressed in tumor-infiltrating MDSCs can facilitate the survival of tumor cells [19]. Moreover, signaling pathways triggered by two types of TNFRs can exhibit mutually synergistic or antagonistic effects, making the situation more complex still [14]. Therefore, further in-depth studies are required.

## 3. The Introduction of MDSCs

Immune responses mediated by immune cells derived from bone marrow hematopoietic stem cells have gradually evolved to be the main mechanisms by which a host organism resists infections [20]. However, there is also a population of immunosuppressive cells named MDSCs in individuals. In healthy individuals MDSCs, present in low numbers in the circulation, are involved in the regulation of immune responses and tissue repair [21]. However, during immunological responses to infections, inflammation and cancer, an increased population of bone marrow cells differentiate to MDSCs so that this population rapidly expands [21]. MDSCs have similar phenotypes, with normal neutrophils and monocytes, but they exert immunosuppressive functions [22]. A substantial body of evidence suggests that MDSCs play critical roles in immunosuppression, angiogenesis, drug resistance, and the metastasis of tumors [21]. Researchers have also found that the immunosuppression of MDSCs is involved in cases of chronic inflammation, pregnancy, autoimmune diseases, infections, and transplantation [20,23]. Therefore, the targeting of MDSCs has important clinical implications.

Classical activation of immune cells occurs when the body responds to a strong signal stimulated by a pathogen, such as an acute infection or trauma. At this point, neutrophils and monocytes are rapidly mobilized, phagocytosis is enhanced, major histocompatibility complex class II (MHCII) and co-stimulatory molecules are upregulated, and anti-inflammatory factors such as TNF-α are produced. However, in some chronic diseases, such as chronic infection and cancer, the signals stimulated by the pathogen are weak and of long duration. In such cases, inflammatory factors and endoplasmic reticulum stress are activated. Neutrophils and monocytes now exhibit heterogeneous and immature phenotypes. They exhibit weaker phagocytic activity, and produce more Arg1, ROS, and NO. These pathologically activated cells are known as MDSCs and have specific cell surface markers CD11b and GR1 [20]. Current research suggests that MDSCs consist of two major isoforms: granulocytic MDSCs (G-MDSCs), and monocytic MDSCs (M-MSDC). G-MDSCs have similar phenotypes to neutrophils and are defined as CD11b+Ly6G+Ly6C- cells. M-MDSCs have similar phenotypes to monocytes and are defined as CD11b+Ly6G-Ly6C+ cells. In addition, a third population of MDSCs, known as early MDSCs (eMDSCs), have been identified in humans. These exhibit colony-forming activity. However, this population of MDSCs has not yet been defined in mice [24]. In mice, the amount of M-MDSCs is lower than G-MDSCs [25]. However, M-MDSCs exhibit a stronger immunosuppressive capacity than G-MDSCs [24]. In general, M-MDSCs exert their immunosuppressive functions by means of iNOS, Arg1 and some anti-inflammatory factors, while the immunosuppressive functions of G-MDSCs are mediated by ROS [26]. Given the unique biological features of MDSCs, and their important roles in inflammation and the immune system, the targeting of MDSCs offers new potential opportunities for the treatment of cancers, chronic inflammatory diseases, and some autoimmune disorders.

## 4. The Role of TNF Signaling on MDSCs

Prolonged exposure to TNF-α can suppress the immune systems of animals [27]. In addition to promoting the expansive proliferation, phenotypic stability, and enhanced suppressive capacity of Tregs, TNF-α also affects the recruitment and function of MDSCs [28,29]. Both TNFR1 and TNFR2 are expressed in MDSCs. Although the levels of TNFR2 expression are much higher than those of TNFR1, the TNF signaling pathways mediated by both TNFR1 and TNFR2 play important roles in the accumulation and function of MDSCs [30].

### 4.1. The Effects of TNF Signaling in the Accumulation of MDSCs

The effects of TNF-α may be crucial for the accumulation of MDSCs in many pathological processes, including tumor growth and chronic inflammation. For example, the TNF signaling promotes the accumulation of MDSCs in the chronic inflammation and carcinogenesis induced by 3-methylcholanthrene (MCA) [13]. However, the effects of TNF signaling pathways in cases of acute pleurisy induced by mycobacterium bovis appear to be different [31]. Compared with WT mice, MDSCs accumulate abundantly in TNF knockout (KO) mice but not in tmTNF knock-in (KI) mice, indicating that tmTNF-α suppresses the expansion of MDSCs. Moreover, M-MDSCs and G-MDSCs isolated from TNF KO mice cannot suppress the proliferation of T cells [31]. We explain these phenomena thus: MDSCs may differentiate into mature immune cells in acute inflammation. For this reason, the cells detected in [31] may have been mature immune cells equipped with the same phenotypes as MDSCs. By reviewing other studies, we will further articulate the influence of TNF-α on the accumulation of MDSCs in terms of their development, differentiation, apoptosis, and induction.

#### 4.1.1. TNF Signaling Promotes the Development of MDSCs and Suppresses Their Differentiation

**Figure 1:**
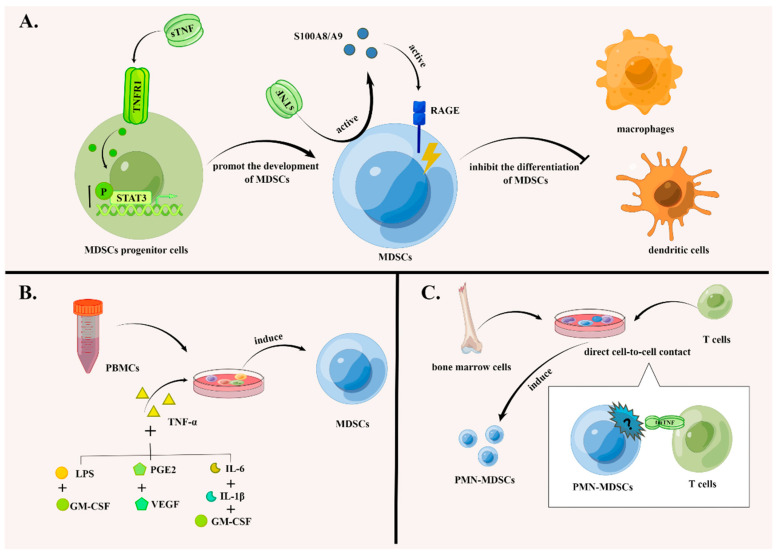
TNF signaling promotes the accumulation of MDSCs in multiple ways. (**A**) TNF signaling promotes the development of MDSCs and suppresses their differentiation. sTNF-α enhances the phosphorylation of transcription factors of Stat3 in MDSCs progenitor cells, which facilitates the development of MDSCs. In addition, TNF-α inhibits the differentiation of MDSCs into dendritic cells and macrophages mediated by the inflammatory proteins S100A8/A9 and their corresponding receptors, such as RAGE, promoting the accumulation of MDSCs. (**B**) MDSCs can be induced from PBMCs with the participation of TNF-α. The combined administration of TNF-α, GM-CSF and LPS; the administration of TNF-α, PEG2 and VEGF; and the administration of TNF-α, IL-6, IL-1β and GM-CSF can induce MDSCs with immunosuppressive functions from PBMCs. (**C**) PMN-MDSCs can differentiate from bone marrow cells. PMN-MDSCs can be induced from bone marrow by CD4+ T cells depending on direct cell-to-cell contact and tmTNF-α signal transduction. The figure was created using Figdraw (https://www.figdraw.com/ accessed on 19 August 2022).

The authors [13] found that sTNF-α enhances the phosphorylation of Stat3 in a cancer model induced by 3-methylcholanthrene, facilitating the development of MDSCs and leading to the accumulation of MDSCs. Feldman et al. came to the conclusion that TNF-α inhibits the differentiation of MDSCs into dendritic cells and macrophages mediated by inflammatory proteins S100A8/A9 and their corresponding receptors, thereby promoting the accumulation of MDSCs [32]. As MDSC progenitor cells lack TNFR2 and express TNFR1 only, Andrea et al. argued that the aforementioned mechanisms are mediated by sTNF-α/TNFR1 signaling [13] (Figure 1).

#### 4.1.2. TNF Signaling Promotes the Induction of MDSCs In Vitro

As an important kind of immunosuppressive cells, MDSCs exert considerable therapeutic effects in cases of autoimmune disease, organ transplantation, and cardiovascular disease [33]. For this reason, the in vitro induction of MDSCs has become area of intense scientific research.

MDSCs can be induced from PBMCs with the participation of sTNF-α. Hsu et al. found that the combined administration of sTNF-α, GM-CSF and LPS induced MDSCs with an immunosuppressive function from PBMCs [34]. Seledtsov et al. found that, in combination with PEG2 and VEGF, sTNF-α-induced MDSCs from PBMCs [35]. PBMCs also differentiated into MDSCs after the combined administration of pro-inflammatory factors TNF-α, IL-6, IL-1β, and GM-CSF [36,37] (Figure 1).

MDSCs can also differentiate from bone marrow cells. The authors [38] reported that PMN-MDSCs were induced from CD33+ bone marrow by CD4+ T cells, in a process dependent upon direct cell-to-cell contact and tmTNF-α signal transduction. In summary, we can state that TNF-α participates in the induction of MDSCs through multiple pathways (Figure 1).

In addition, MDSCs can differentiate from bone marrow cells. It has been reported that PMN-MDSCs can be induced from CD33+ bone marrow by CD4+ T cells, which depend on direct cell-to-cell contact and tmTNF-α signal transduction [38]. Taken together, TNF-α participates in the induction of MDSCs through multiple pathways (Figure 1).

#### 4.1.3. TNF Signaling Inhibits the Apoptosis of MDSCs

**Figure 2:**
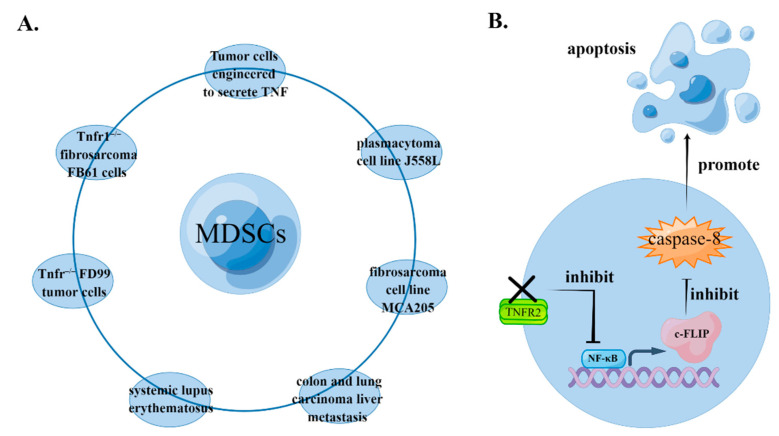
TNF signaling inhibits the apoptosis of MDSCs. (**A**) In systemic lupus erythematosus, colon and lung carcinoma liver metastasis models, transplanted tumor models with Tnfr−/− FD99 tumor cells, Tnfr−/− fibrosarcoma FB61 cells, tumor cells engineered to secrete TNF, plasmacytoma cell line J558L and fibrosarcoma cell line MCA205, TNF signaling inhibits the apoptosis of MDSCs. (**B**) In TNFR2-deleted MDSCs, NF-κB signaling and its downstream molecule c-FLIP are downregulated, and the activity of caspase-8 is then increased, promoting the apoptosis of MDSCs. The figure was created using Figdraw (https://www.figdraw.com/ accessed on 19 August 2022).

TNF signaling can inhibit the apoptosis of MDSCs in a variety of pathological models, by promoting the accumulation of MDSCs. Qin et al. found spontaneous suppression in the growth of transplanted tumors in Tnfr−/− mice [39]. Zhao et al. found that spontaneous tumor suppression is caused by the absence of TNFR in host cells rather than in transplanted tumors, which is associated with MDSC recruitment in peripheral tissues. In MDSCs purified from spleens of Tnfr−/− mice, NF-κB’s signaling and its downstream molecule cellular FLICE-inhibitory protein (c-FLIP) are downregulated, but activity of caspase-8 increases, suggesting that increased cell apoptosis is the main reason for the decreasing quantity of MDSCs in Tnfr−/− mice. Because there are two types of TNFR, signals mediated by TNFR2 are necessary and sufficient to protect MDSCs from apoptosis, while TNFR1 deletion has no significant effect on tumor growth, MDSC recruitment, the NF-κB signaling pathway, or induction of apoptosis [40]. Moreover, Ham et al. used a liver metastasis model, which indicated recruitment of MDSCs prior to tumor metastasis in the liver, thus contributing to the formation of a tumor microenvironment. TNFR2 plays important roles in this process because the proportion of apoptotic MDSCs is significantly increased in Tnfr2−/− mice [41] (Figure 2).

Estrogen also plays important roles in the anti-apoptotic effects mediated by TNF signaling in MDSCs. Dong et al. found that the proportion of peripheral MDSCs is higher in women than men with systemic lupus erythematosus (SLE). The authors [42] found that 17β-estradiol significantly inhibited MDSC apoptosis by secreting TNF-α, promoting the accumulation of MDSCs in peripheral blood. In addition, sexual dimorphism has been observed in the regulation of the immune microenvironment in liver metastasis. In cases of liver metastasis from colon cancer or lung cancer, for example, the accumulation of MDSCs is TNFR2-dependent in females but not in males. The signaling pathways mediated by estrogen and its receptor induce the expression of TNFR2 in the immune microenvironment of the liver. Then, by competing with TNFR1, the signaling pathways mediated by TNFR2 protect MDSCs from the injury of ‘death signals’ in the TNF-α-rich immune microenvironment of the liver, and this contributes to the survival and immunosuppressive activity of MDSCs [43].

In addition to tmTNF-α/TNFR2 signals, the signaling pathways mediated by sTNF can also alleviate apoptosis in MDSCs. By inducing the activation of NF-κB, upregulating the expression of cFLIP, and inhibiting the expression of caspase-8, sTNF protects MDSCs from apoptosis and prolongs their survival [40]. However, TNFR2 may also be involved in this process. Specifically, if TNFR1 is ubiquitously expressed, sTNF-α can selectively trigger cell survival signaling mediated by TNFR1 alone. However, if TNFR2 is ubiquitously expressed, the aforementioned cell survival signaling can be cross-transduced and amplified through signaling mediated by TNFR2 [13].

These findings indicate that the apoptosis of MDSCs can be profoundly influenced by TNF-α signaling, and this is likely due to a combination of factors. The ability of MDSCs to inhibit T cell proliferation is well known. Deng et al. studied the interaction between T cells and MDSCs and found that CD8+ T cells induce the apoptosis of MDSCs in a TNF-dependent manner. For this reason, a combined treatment of high-dose ionizing radiation and anti-PD-L1 can downregulate the accumulation of MDSCs by potentiating the activation of CD8+ T cells, which promotes tumor regression to a certain extent [44].

We can now summarize the mechanisms by which TNF signaling pathways facilitate the accumulation of MDSCs, through promoting their development, inhibiting their differentiation and apoptosis, contributing to their induction, etc. Most of the aforementioned mechanisms are mediated by tmTNF-TNFR2. This may be because expression of TNFR2 is much higher than that of TNFR1 in MDSCs. sTNF also plays important roles in the accumulation of MDSCs. During the early stages of tumorigenesis, MDSCs exhibit low levels of expansion. However, MDSC expansion greatly increases with advanced tumor progression. This phenomenon may be explained by the fact that MDSCs are regulated by various kinds of cytokines at different stages of the carcinogenesis process. Specifically, IL-1α may regulate the growth of MDSCs during the initiation of carcinogenesis, while VEGF and GM-CSF promote the expansion of MDSCs after tumor formation [21,45]. However, sTNF may be involved in the regulation of MDSCs throughout the carcinogenesis process and play central regulatory roles [21,32,40,45]. For this reason, the TNF signaling pathways mediated by sTNF in MDSCs are an important subject for future research.

### 4.2. The Effects of TNF-α Signaling in the Function of MDSCs

The interactions of inflammation and its regulatory pathways contribute to an effective immune response that eradicates pathogens while avoiding excessive damage to host cells. MDSCs are a kind of immunosuppressive cells. They help to maintain the immune homeostasis of the body by inhibiting excessive immune response [29]. Systemic TNF inhibition affects the suppressive functions of MDSCs, according to the authors [9].

#### 4.2.1. MDSCs Suppress the Effect of T Cells through tmTNFα-TNFR2 Signaling

**Figure 3:**
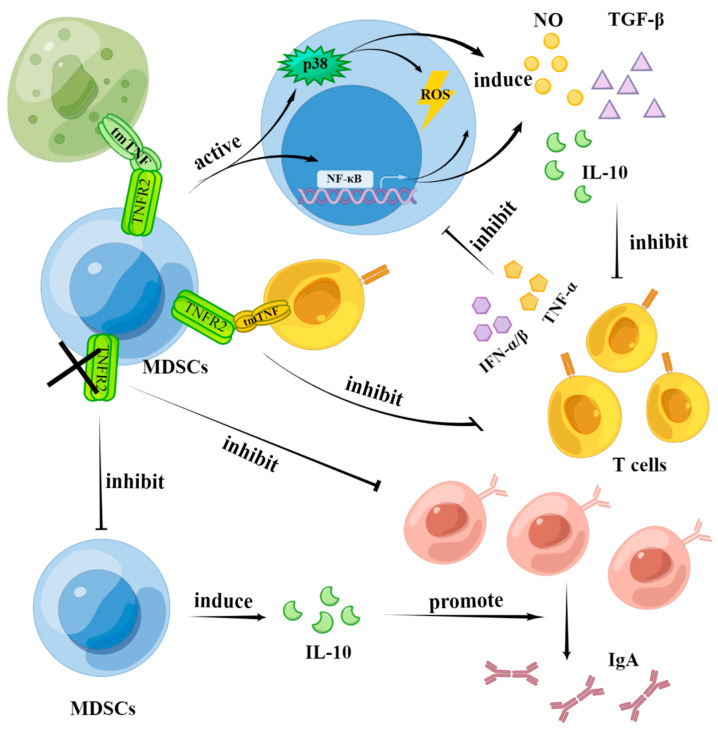
The effects of TNF signaling in the function of MDSCs. TNF signaling pathways enhance the inhibitory effect of MDSCs on T cells. tmTNF activates the immunosuppressive function of MDSCs by activating NF-κB and p38 signaling pathways mediated by TNFR2. By secreting immunosuppressive mediators including NO, ROS, IL-10 and TGF-β, MDSCs inhibit the proliferation and function of T cells. Activated-memory T cells then inhibit the immunosuppressive function of MDSCs by secreting TNF-α and IFN-α/β. Additionally, tmTNF in MDSCs interacts with TNFR2 in CD4+ T cells. This is necessary for the inhibitory effect of MDSCs on CD4+ T cells. In terms of B cells, a specific blockade of TNFR2 in MDSCs can inhibit cell proliferation. Finally, MDSCs can secrete IL-10 in a TNFR2-dependent manner, promoting the activation of B cells and the production of IgA. The figure was created using Figdraw (https://www.figdraw.com/ accessed on 19 August 2022).

Impaired T-cell immunity during chronic inflammation is mediated by MDSCs. Once MDSCs are depleted, the immune function of T cells is fully restored [32]. Signaling pathways mediated by TNFR2 in MDSCs play important roles in suppressing the function of T cells [46]. Hu et al. found that ectopic expression of tmTNF in tumor cells enhances the inhibitory activity of MDSCs. They incubated MDSCs from tumor-bearing mice with sTNF, tmTNF, or their neutralizing antibodies, and found that tmTNF-α activated MDSCs with enhanced suppressive activities, including upregulating arginase-1 and inducible NO synthase transcription, promoting secretion of NO, ROS, IL-10, and TGF-β, and enhancing the inhibition of lymphocyte proliferation. However, sTNF-α seems to have little influence on the immunosuppressive function of MDSCs. Moreover, studies on MDSCs in Tnfr1–/– and Tnfr2–/– tumor-bearing mice have shown that tmTNF requires TNFR2 to mediate the activation of NF-κB and p38 signaling pathways, which activate the immunosuppressive function of MDSCs [30]. The authors [27] used an inflammation model and found that TNFR2 is required for MDSCs to exert their immunosuppressive function. When TNFR2 is lacking. The ability of MDSCs to inhibit the proliferation of T cells is impaired, and this may contribute to the impaired production of NO in MDSCs (Figure 3).

Feldman et al. found that TNF-α enhanced the immunosuppressive function of MDSCs in a chronic inflammation model using heat-inactivated pathogens. They also found downregulation of the T-cell antigen receptor ζ chain, dysfunction of T cells, and the appearance of NK cells in vivo [32]. In addition to the effects of exogenous TNF, the own expression of TNF in MDSCs also has important impacts upon immunosuppressive functions [31]. The authors [32] demonstrated that the production of ROS, the expression of iNOS, the bioactivity of Arg1 and the ability to inhibit T cell proliferation all decrease significantly in MDSCs isolated from Tnf−/− mice.

Chavez-Gala et al. constructed a mouse model with overexpression of tmTNF. Two subtypes of MDSCs (M-MDSCs and G-MDSCs) were isolated from the tmTNF KI mice and cocultured with TNFR1 or TNFR2 knockout CD4+ T cells, respectively. Compared with the wild-type (WT) mice, the M-MDSCs and G-MDSCs obtained from tmTNF KI mice promoted the proliferation of TNFR2 knockout CD4+ T cells. However, in the case of TNFR1 knockout CD4+ T cells, two subtypes of MDSCs from tmTNF KI mice continued to inhibit proliferation and exhibited a stronger inhibitory effect, compared with those obtained from WT mice. The inhibitory effects of MDSCs on CD4+ T cells, therefore, require the self-expression of tmTNF in MDSCs and TNFR2 in CD4+ T cells [31].

#### 4.2.2. MDSCs Enhance the Activation and Proliferation of B Cells through TNFR2 Signaling 

Compared with T cells, there have been fewer studies concerning the effects of MDSCs on B cells. The authors [47] reported the rejection of spontaneous mammary adenocarcinoma in B cell-deficient mice. In contrast, the authors [48] found that adoptively transferred B cells or serum can facilitate the development of squamous cell carcinoma. These studies show that B cells may promote tumor development in some cases. Xu et al. also demonstrated that a specific blockade of TNFR2 inhibits the proliferation of B cells. MDSCs express IL-10 in a TNFR2-dependent way, facilitating the activation of B cells and the production of IgA, thereby promoting tumor development [49] (Figure 3).

#### 4.2.3. T Cells Affect the Function of MDSCs through TNF Signaling

The immunosuppressive effects of MDSCs may stem from a physiological regulation process involving negative feedback from T cells. MDSCs and T cells influence each other and, thus, act together to maintain immune homeostasis in organisms. 

The authors [50] found that activated T cells inhibit the function of MDSCs by secreting IFN-α/β and TNF-α, and the role of TNF-α is also important (Figure 3). However, T cells can induce MDSCs and prolong their survival, and this can be understood as the negative feedback regulation of T cells themselves. The different results obtained in different studies might be attributed to the different activation states of T cells [38].

Regarding the immunosuppressive function of MDSCs regulated by TNF signaling pathways, researchers have found that sTNF mediates the acquisition and maintenance of immunosuppressive function in MDSCs, while tmTNF enhances the immunosuppressive function of MDSCs [30,32] (Figure 3). In addition, the crosstalk between NK cells and dendritic cells mediated by MDSCs is sTNF-dependent [32]. In conclusion, the immunosuppressive function of MDSCs may be a consequence of basal inhibition activity dependent on sTNF and enhanced inhibition activity mediated by tmTNF [51].

### 4.3. The Effects of TNF Signaling Pathways in the Chemotaxis of MDSCs 

In one study of lung cancer, researchers found that the recruitment of MDSCs decreased in TNF KO mice compared with WT mice, inhibiting tumor growth. Ba et al. conducted studies of Tnfr−/− and WT mice injected subcutaneously with murine hepatic carcinoma cell line H22 cells. They found that TNFR deletion inhibits the accumulation of M-MDSCs and G-MDSCs in spleens and tumor tissue but has no influence on their prevalence in bone marrow. Moreover, the chemotactic ability of TNFR-null MDSCs is weakened because their expression of chemokines such as CXCL12 and CXCR4 decreases obviously [52].

**Figure 4:**
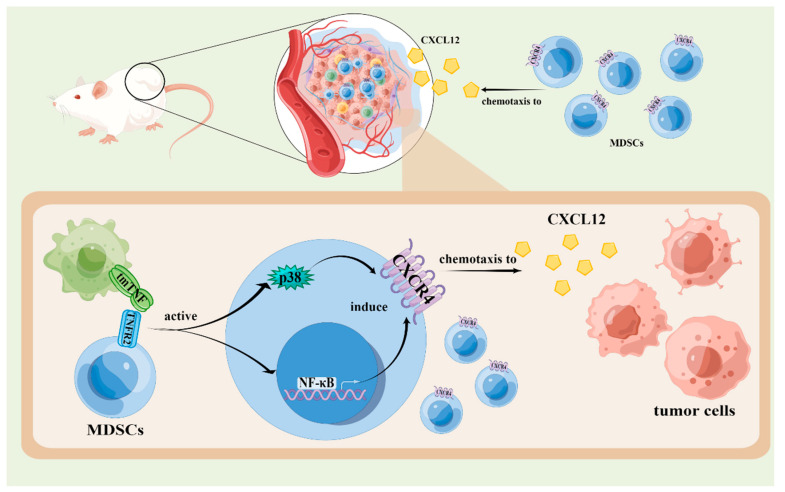
The effects of TNF signaling on the chemotaxis of MDSCs. The tmTNF activates NF-κB and p38 signaling pathways in MDSCs mediated by TNFR2, inducing their expression of CXCR4, and subsequently enhancing the chemotaxis of MDSCs in tumor tissues secreting CXCL12. The figure was created using Figdraw (https://www.figdraw.com/ accessed on 19 August 2022).

In another study, researchers intervened Tnfr1–/– and Tnfr2–/– mice with sTNF and tmTNF, respectively. They found that tmTNF activates NF-κB and p38 signaling pathways in MDSCs mediated by TNFR2, inducing their expression of CXCR4, and subsequently enhancing the chemotactic ability of MDSCs (Figure 4). In tumor growth, CXCR4 is a major contributor to the recruitment of MDSCs. The inhibited expression of CXCR4 is one of the significant factors in tumor regression promoted by TNFR deletion [52].

In this review, we have highlighted the key roles played by TNF-α and TNFR in the accumulation, immunosuppressive function, and chemotaxis of MDSCs. We have analyzed their importance for the control of inflammation and tumor progression, and discussed the potential targeting effect of these signaling pathways, thus providing new ideas for the immunotherapeutic treatment of health disorders and disease.

## 5. The Potential Therapeutic Effects of TNF Signaling Pathways in MDSCs

TNF-α is a potent pro-inflammatory factor and exerts pleiotropic effects in a variety of cell types. TNF-α plays critical roles in some tumors or chronic inflammatory diseases by influencing MDSCs, which are an important population of immunosuppressive cells. For this reason, researchers are now focusing on regulating TNF-α or its signaling pathways in MDSCs with the objective of developing new disease treatments. Several recent studies are summarized in Table 1.

The data presented in this table are all from preclinical studies. In this review, we summarized the pharmacological and biological agents targeting TNF signaling, which have promising therapeutic perspectives by regulating the recruitment and immunosuppressive function of MDSCs. Although TNF inhibits the apoptosis of MDSCs, it is not the most efficient member of the family to trigger apoptosis. Signaling through Fas, TRAIL-R1 and TRAIL-R2 has been well characterized [57]. Engagement of these receptors delivers a powerful and rapid proapoptotic signal through a death domain-mediated recruitment of the adaptor protein FADD and the formation of the so-called death-inducing signaling complex [58]. Future studies should further examine this and focus on whether TNF plays therapeutic roles through inducing the apoptosis of MDSCs.

Furthermore, there are also some pharmacological agents that target TNF-α, such as adalimumab, golimumab and trastuzumab, some biological agents that target sTNF/TNFR1, such as TNFR1-selective antagonist R1antTNF and TNFR1-specific antibody DMS5540, and some agents that target tmTNF/TNFR2, such as TNFR2-specific neutralizing antibody, and CD3-specific antibody hOKT3γ1(Ala-Ala) [29]. The effects of these agents in MDSCs also need further exploration.

## 6. Discussion 

TNF-α can be seen as a double-edged sword. On the one hand, TNF-α can maintain the host defense function and the immune homeostasis. On the other hand, it is involved in the pathological process of malignant diseases, autoimmune diseases, and inflammatory disorders [8,18]. Although TNF-α is called tumor necrosis factor, it can also promote tumor development. These dual opposite effects of TNF-α may be due to the carcinogenic factors, histologic types of primary tumors, and the local tumor microenvironments, but the detailed mechanisms are still unclear. Moreover, TNF signaling plays important roles in regulating the biological roles of MDSCs, Treg cells, and some other immune cells, which affects the survival of tumor cells. In addition, signaling mediated by two forms of TNF receptors exert their effects cooperatively or antagonistically, making the situation more complex [14].

TNF-α is widely expressed throughout the body and mediate wide-ranging physiological roles. Therefore, cancer risk and other serious side effects should be monitored when using TNF-α or its inhibitors for treatment [59,60]. In this review, we discussed the regulation of signaling pathways mediated by TNFR on MDSCs in detail. According to the specific types of diseases, topical drug administration or adjunctive medication in a specific time window may alleviate the adverse effects of the treatment on the body and produce better therapeutic effects. Therefore, more studies are needed to elucidate the specific mechanisms whereby TNF-α exerts these effects.

In conclusion, our review summarized the regulation and mechanisms of TNF signaling on the recruitment, immunosuppressive function, and chemotaxis of MDSCs in certain tumors, chronic inflammatory diseases, and some autoimmune disorders. Regulating TNF-α signaling pathways in MDSCs may be another therapeutic strategy, which provides new ideas and directions for future studies.

## Figures and Tables

**Table 1:** The therapeutic roles of TNF signaling pathways in MDSCs to some diseases.

Disease Models	Pharmacological or Biological Agents	Mechanisms	Reference
Melanoma	Decitabine (DCA): A DNA methyltransferase inhibitor.	DAC activates the intrinsic TNF-α-RIP1signaling pathway (an apoptosis pathway) in myeloid-derived suppressor cells (MDSCs), inhibiting the accumulation of MDSCs and enhancing the sensitivity of the tumor to immune checkpoint blockade (ICB) therapy.	[53,54]
3-methylcholanthrene (MCA)-induced tumor	XPro1595: A dominant-negative tumor necrosis factor (DN-TNF) and specific antagonist of sTNF. It inactivates endogenous sTNF by forming hetero trimers.	Treatment of XPro1595 can decrease the phosphorylation of STAT3 in MDSCs, inhibiting the recruitment and immunosuppressive function of MDSCs in MCA-induced tumor, delaying the development of the tumor.	[13,29,55]
Fibrosarcoma	Infliximab: A kind of purified recombinational DNA-derived chimeric monoclonal anti-TNF that specifically binds to TNF and blocks its interaction with cell surface receptors.	MDSCs treated with infliximab produce less NO and cannot inhibit the proliferation of T cells. By downregulating the recruitment and immunosuppressive function of MDSCs, infliximab suppresses the growth of tumors.	[9]
Fibrosarcoma	Etanercept: An engineered recombinant protein that comprises of TNFR2 and the fragment crystallizable (Fc) portion of human IgG1. It can specifically bind to TNF and block its interaction with cell surface receptors, downregulating the recruitment and immunosuppressive function of MDSCs	Etanercept inhibits the expression of IL-10 and TGF-β in MDSCs, impairing their immunosuppressive function. Moreover, the expression of ADAM17 in MDSCs decreases after the treatment of etanercept. This gene encodes TACE that cuts CD26L in the surface of T cells, preventing naive T cells from migrating to the inflammation site and inhibiting their activation. Therefore, etanercept suppresses tumor growth by downregulating the recruitment and immunosuppressive function of MDSCs.	[9]
Benzo(a)pyrene-induced chronic pneumonia		By downregulating the recruitment and immunosuppressive function of MDSCs, etanercept prevents or delays the malignant transformation of chronic inflammation induced by benzo(a)pyrene.	[56]
Inflammation induced by bacille Calmette-Guerin (BCG)		Etanercept promotes the differentiation of MDSCs to mature dendritic cells and macrophages and inhibits the immunosuppressive function of MDSCs by decreasing the generation of NO and ROS, which reconstructs the activity of NK cells and T cells and promotes the recovery of immune function in organisms.	[32]

## Data Availability

Not applicable.
